# Potentials of *Gynura procumbens* to modulate chronic stress and immunological responses in *Oreochromis niloticus*

**DOI:** 10.1371/journal.pone.0295137

**Published:** 2023-12-27

**Authors:** Sinthia Kabir Mumu, Nahian Fahim, Eaint Honey Aung Win, Kusum Parajuli, Lindee Mason, Isaac Wendel, Ahmed Mustafa

**Affiliations:** 1 Department of Biological Sciences, Purdue University Fort Wayne, Fort Wayne, IN, United States of America; 2 University of California Los Angeles, Los Angeles, CA, United States of America; Tanta University Faculty of Agriculture, EGYPT

## Abstract

Natural products and traditional remedies have become more popular over the years since they have less harmful side effects and are considered environmentally friendly. In this study we aimed to investigate the potential of *Gynura procumbens* extract (GPE), a well-known traditional medicinal plant extract, on the stress modulation of *Oreochromis niloticus* (Nile tilapia). Four different experimental groups: control, stress, prevention, and treatment were monitored for 12 weeks. Hydrocortisone (0.01% of body weight) was incorporated with the feed to induce the stress response in stress, prevention and treatment groups. Feed was also supplemented with 0.15% GPE of body weight for the prevention and treatment groups. Cortisol concentration was reduced significantly in the prevention (1870.52 pg/mL; *p* = 0.006) and treatment (2925.91 pg/mL; *p* = 0.002) groups than the stress group (7614.22 pg/mL). The result is substantiated by significant decrease in blood glucose level in prevention (29.5 mg/dL; *p* = 0.002) and treatment (31.5 mg/dL; *p* = 0.006) groups, compared to stress group (47.33 mg/dL) at the end of the experiment. Considering the current finding, we can conclude the GPE has potential to be used as therapeutic option for stress regulation however there is a room for further detailed study to understand the in-depth mechanism.

## Introduction

Fish farming for human consumption is a thriving industry, and with increased interaction with captive fish populations, there is a growing interest in determining how to provide good welfare for the fish we farm [1]. Stress in fish is a significant issue in aquaculture because extensive husbandry techniques such as netting, weighing, sorting, vaccination, and transportation contribute to decreased production efficiency [2,3]. Fish farmers are gradually recognizing the signs of stress since survival, growth, and reproduction among other factors, have been shown to decrease in poor husbandry conditions [4]. Fish physiological responses to environmental stressors have been largely classified as primary and secondary. The release of catecholamines from chromaffin tissue represents the primary stress response, which subsequently initiates the initial neuroendocrine responses [5], and stimulation of the hypothalamic-pituitary-interrenal (HPI) axis, which results in the release of corticosteroids into the bloodstream [6–8]. Essentially, the primary response is the perception of a stressor that involves the activation of a neuroendocrine cascade response involving the secretion and synthesis of cortisol and related compounds, as well as catecholamines such as adrenaline and noradrenaline [6]. In lamprey, the main corticosteroid responding to stress is 11-deoxycortisol, whereas in elasmobranchs it is 1a-hydroxycorticosterone and cortisol, as well as other related steroids in chondrostean and teleosts [9,10]. Hydrocortisone, a cortisol metabolite, is also found in high concentrations in the blood of stressed fish [11].

Secondary stress responses include cardiovascular and respiratory responses, both of which increase the distribution of oxygen and energy substrates into the circulation and adrenaline alters blood flow patterns and permeability within the gills, which affects the osmotic balance [12]. Changes in plasma and tissue ion and metabolite levels, hematological characteristics, and heat shock or stress proteins (HSPs) are all related to physiological changes. This includes metabolic reactions, respiration, pH concentration, hydromineral stability, immunity, and cellular responses [8,13]. Another response to stress hormones is immunosuppression. Immunosuppression signifies many tertiary stress responses which are maladaptive. These responses include decreases in disease resistance, reproduction, growth, learning, and other behaviors like predator avoidance are all hampered [14]. Apart from technological and infrastructure changes, implementing new feeding strategies is a straightforward and practical way to improve fish welfare. In this context, the concept of functional food (food with beneficial effects on the organism that are not nutritional in nature) has emerged as a new method to improve overall health, including welfare [15].

*Gynura procumbens*, also known locally as “longevity spinach” or "*Sambung Nyawa*," is mainly grown for medicinal purposes in Southeast Asia, especially in Indonesia, Malaysia, and Thailand. It is a member of the Asteraceae family and has been used as both a vegetable and a medicine for several years. As a traditional medicinal plant, the leaves are commonly used to treat a variety of diseases caused by oxidative stress, including inflammation, cancer, diabetes, hypertension, and hyperlipidemia [16]. The extract of *Gynura procumbens* (GPE) has been shown in prior pharmacological research to have anti-inflammatory, antihypertensive, antihyperlipidemic, antioxidative, and cardioprotective attributes [17]. Several organic compounds, including syringic acid, quercetin, N, N-dimethylanthranilic acid, dehydrovomifoliol, β-sitosterol 3-O-β-D-glucopyranoside, schottenol, and montanic acid, were isolated and their structures determined. The results of the tests revealed that the *Gynura procumbens* extract (GPE) were active as glucosidase inhibitors, which could help with diabetes treatment [18]. Several studies have found that bioactive components such as rutin, astragalin, quercetin, kaempferol, kaempferol-3-O-rutinoside, and kaempferol-3, extracted from *Gynura procumbens* with ethanol help lower blood sugar levels in a variety of rodent studies [19,20]. Based on previous research showing that GPE can reduce glucose levels, the main biomarker of stress, we evaluated the consequence of GPE on lessening the chronic stress response of Nile tilapia in this study. The present result demonstrated the significantly (p<0.05) low level of cortisol and blood glucose in the treatment and prevention group compared to the stressed group. This encouraging finding implies that the GPE could be the potential source of phytotherapeutics for stress management in aquaculture and also opens up the direction for further studies.

## 2. Material and methods

### 2.1 Preparation of extract

Leaf extract section of this study followed by our previous paper [21]. Following the collection, cleansing, and mashing of the GPE leaves, an extract was made by combining them with a 25% ethanol solution, resulting in a concentration of 0.25g/mL. Leaf extract preparation was slightly modified from our previous study to extract significant amount of biologically active compounds. We filtered and mixed the GPE leaf paste for 24 hours at room temperature on a shaker mixture [22–24] rather than 20 minutes [21]. To prepare the fish feed for the multiple treatment groups, the prepared GPE was used.

### 2.2 Feed preparation

In order to prepare the stress feed, commercial feed was coated with hydrocortisone at a concentration of 0.01% of body weight [25]. According to the experimental design, 0.01% hydrocortisone solution prepared with 25% ethanol, mixed well with the commercial feed, and air-dried it overnight. On the following day, the Gynura extract was made and combined with the dried feed at a concentration of 0.13% of body weight. The mixture was then left to air dry overnight, in preparation for the subsequent two weeks of feeding. Following each biweekly sample, the weight of the fish from each treatment group was determined, and the proper feed was given in accordance with the body weight. Commercial feed was used (Table 1) to prepare the control and stress feeds. Experimental feed then divided into three groups: stress group (feed with hydrocortisone throughout the experiment), prevention group (0.15% GPE treatment with induced stress for the complete experiment) and treatment group (first 6 weeks induced stress with 0.01% hydrocortisone and after 6 weeks started 0.15% GPE treatment). The approximate composition of the groups of control and experimental feed is shown in Table 1.

**Table 1:** The table shows the feed ingredients and formulations that were used for each of the treatment groups.

Ingredients (% feed weight)	Control	Stress	Prevention	Treatment
Crude protein	50	50	50	50
Crude fat	16	16	16	16
Crude fiber	3	3	3	3
Calcium (Ca)	5.2	5.2	5.2	5.2
Phosphorus (P)	1.3	1.3	1.3	1.3
Sodium (Na)	0.6	0.6	0.6	0.6

### 2.3 Fish maintenance

Fish were kept under optimum circumstances (pH: 6.0–7.0; ammonia: 0–3.0 mg/dL; temperature: 25–28°C; dissolved oxygen: 5.00–7.00 mg/L; and photoperiod: 12 h:12 h, light: dark) in recirculating aquaculture system. The initial average weight and length of Nile tilapia in our chronic study were 38±2.45g and 13±0.96 cm respectively. Apart from the quantity of food, the method of fish maintenance was acquired from our previous article [21]. The fish were fed twice daily at 1% of their body weight (a total of 2%/day). While we fed them a total of 3% of their body weight each day during our acute study, we noticed that they did not consume the full 3%, and the leftover food led to an increase in ammonia.

All fish were taken care of following an approved protocol by Purdue University Animal Care and Usage Committee (PACUC) following the guidelines of the US National Research Council’s "Guide for the Care and Use of Laboratory Animals".

### 2.4 Experimental design

**Fig 1:**
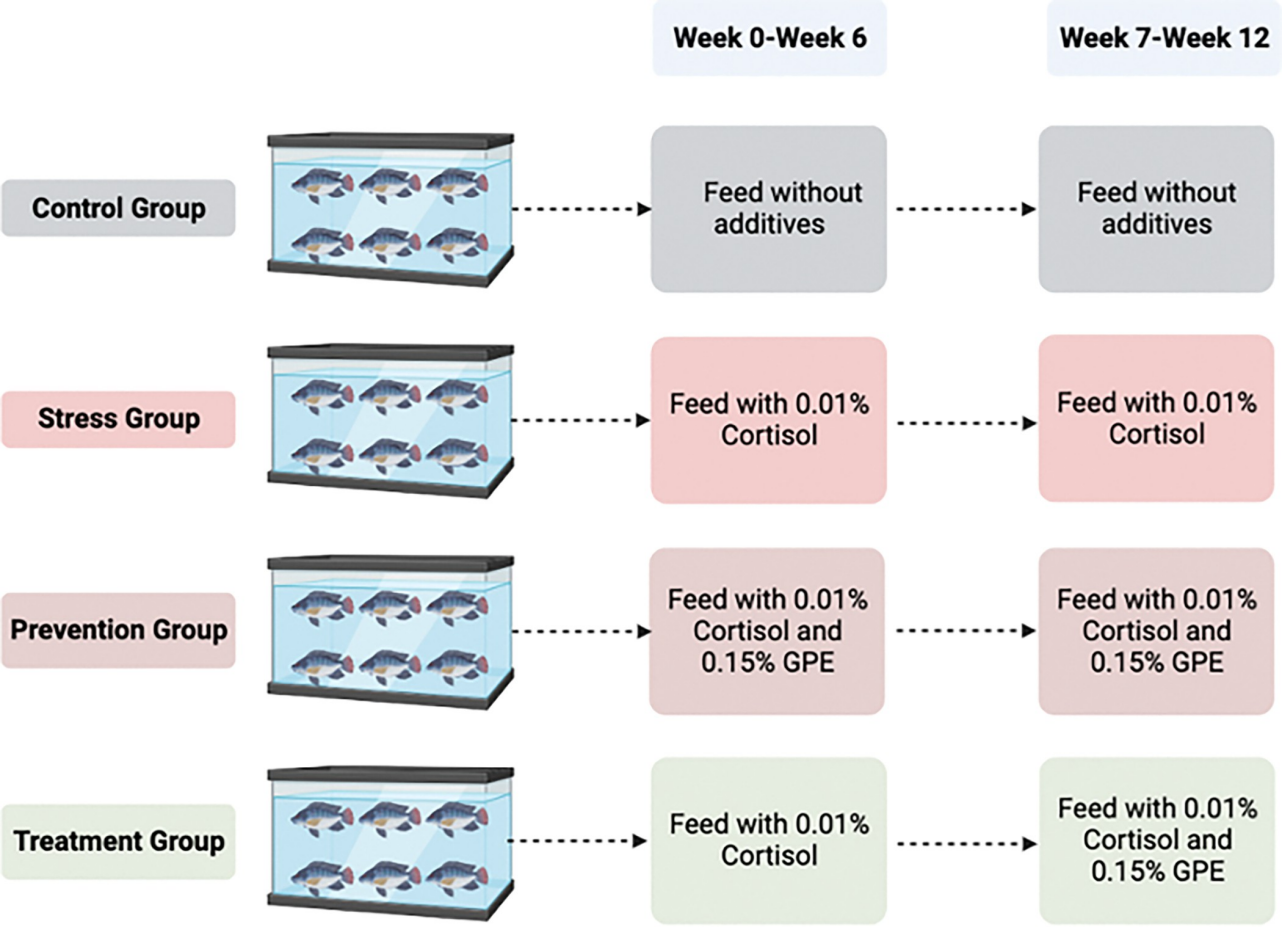
The picture represents experimental design for the chronic study according to the groups and their treatment regime. Cortisol and GPE have been coated on the commercial feed (please review the feed preparation in the method section).

Fig 1 depicts the four groups that made up our experiment. In order to reduce stress in three of the four groups (stress, preventive, and treatment), commercial feed was supplemented with hydrocortisone (0.01% body weight) [25], while the fourth group (non-stress control group) received ordinary commercial feed. During the duration of the experiment, the stress group received hydrocortisone without GPE while the preventative group received GPE plus hydrocortisone-supplemented diet from weeks 0 to 12. However, the treatment group was fed with hydrocortisone feed for the entire experimental period of 0–12 weeks and was treated with GPE from weeks 6–12. The different groups were used to determine the optimal regimen to evaluate the effects of stress on hematological stress biomarkers.

### 2.5 Sampling frequency and parameters

With >200 mg/L of tricaine mesylate, the fish were euthanized. Heparinized syringes were used to draw blood from the caudal vein to prevent clotting. A 1.5 mL Eppendorf tube was used to collect the blood, which was then placed on ice. Everything was completed within two minutes of catching the fish to reduce handling stress. Blood was drawn, and the results were used to calculate blood glucose levels, packed cell volume, and lysozyme activity. The liver and spleen were then removed from the fish using aseptic technique. Additionally, fish length, fish weight, spleen and liver weight were also assessed. Fish were sampled at weeks 0 (baseline), 2, 4, 6, 8, 10, and 12. On the sampling day at each time point, 3 fish for each group were sacrificed in order to measure the parameters. For each group, there was a replicate of 2 tanks and there were 6 samples for each parameter that was collected. (3 X 2 = 6 fish/group, 4 groups).

### 2.6 Cortisol

The serum was obtained from the collected blood by centrifuging the samples at 5000 rpm for 10 minutes. A significant stress marker known as plasma cortisol was measured from plasma. The Cayman Chemical Cortisol ELISA kit (Item No. 500360) (Ann Arbor, MI, USA) was used in accordance with the manufacturer’s instructions to measure the cortisol level in the plasma.

### 2.7 Glucose

Blood glucose level is a useful indicator of acute stress since the release of glucose into the blood is sensitive to stress. Even small alterations can be detected immediately. Blood glucose fluctuations cause an increase in oxygen consumption, a decrease in metabolism, and a substantial reduction in immune function, all of which are manifestations of stress [26]. This experiment used a glucometer to evaluate the blood glucose level (Free-style, Abbott Laboratories, CA) [27].

### 2.8 Packed cell volume

The packed cell volume (PCV) is measured by taking the percentage of red blood cell present in the blood. According to findings from physiological research, stress has an effect on red blood cells, which can be seen as an increase in red blood cell production [28]. The methods of measuring packed cell volume were followed by the previously published article by Mumu and Mustafa, 2022 [21].

### 2.9 Protein

The total plasma protein was measured using a refractometer. A small quantity of serum (1–2 drops) was introduced onto the prism surface of the refractometer using a hematocrit tube. The protein content was then determined by observing the light refraction and reading the value in g/100ml.

### 2.10 Spleen somatic index %

The spleen is a reservoir for lymphocytes and red blood cells before they’re needed by the body’s immune system. Previous research has suggested that stress and the spleen’s somatic index may be related [29]. The spleen somatic index % (SSI), which measures the weight of a fish’s spleen in relation to its total mass, is assessed using the following formula [21]:

$$SSI=\frac{spleen\;weight(g)*100}{body\;weight(g)}$$

### 2.11 Hepatosomatic index %

Glycogen is stored in the fish’s liver and muscles that are massed in the presence of glucagon. Furthermore, the pancreas tends to produce glucagon in high stress conditions and during growth. The liver’s overall size and mass vary as the stored glycogen are converted into glucose and discharged into the bloodstream. Thus, the hepatosomatic index (HSI) is a credible determinant of chronic stress [14]. The fish liver was collected, weighed, and the HSI was calculated as follows:

$$HSI=\frac{liver\;weight(g)*100}{body\;weight(g)}$$

### 2.12 Lysozyme activity

During times of stress, the fight-or-flight response allows organisms to deal with and overcome bacterial invasions. Lysozyme is an antimicrobial enzyme found in plasma. This enzyme can break down the cell wall of microorganisms, particularly Gram-positive bacteria. It is involved in many species’ defense mechanisms, including fish, and was thus chosen for this study [30]. The methodology of lysozyme activity assay (LLA) was described in previously published research (Mumu and Mustafa, 2022) [21]. The LAA was calculated using the following formula:

$$LAA=\frac{\left(Final\;transmittance–Initial\;transmittance\right)}{Total\;elapsed\;time(minute)}$$

### 2.13 Statistical analysis

The Shapiro-Wilk test was used to assess the normality of the data. For normally distributed data, statistical analysis was performed using SigmaPlot 14.5, with a one-way ANOVA and Tukey’s test for post hoc analysis (Systat Software, San Jose, CA). If the data was not normally distributed, alternative non-parametric Kruskal-Wallis test was considered. The results are presented as means ± SD, and statistical significance was defined as a p-value less than 0.05.

## Results

**Fig 2:**
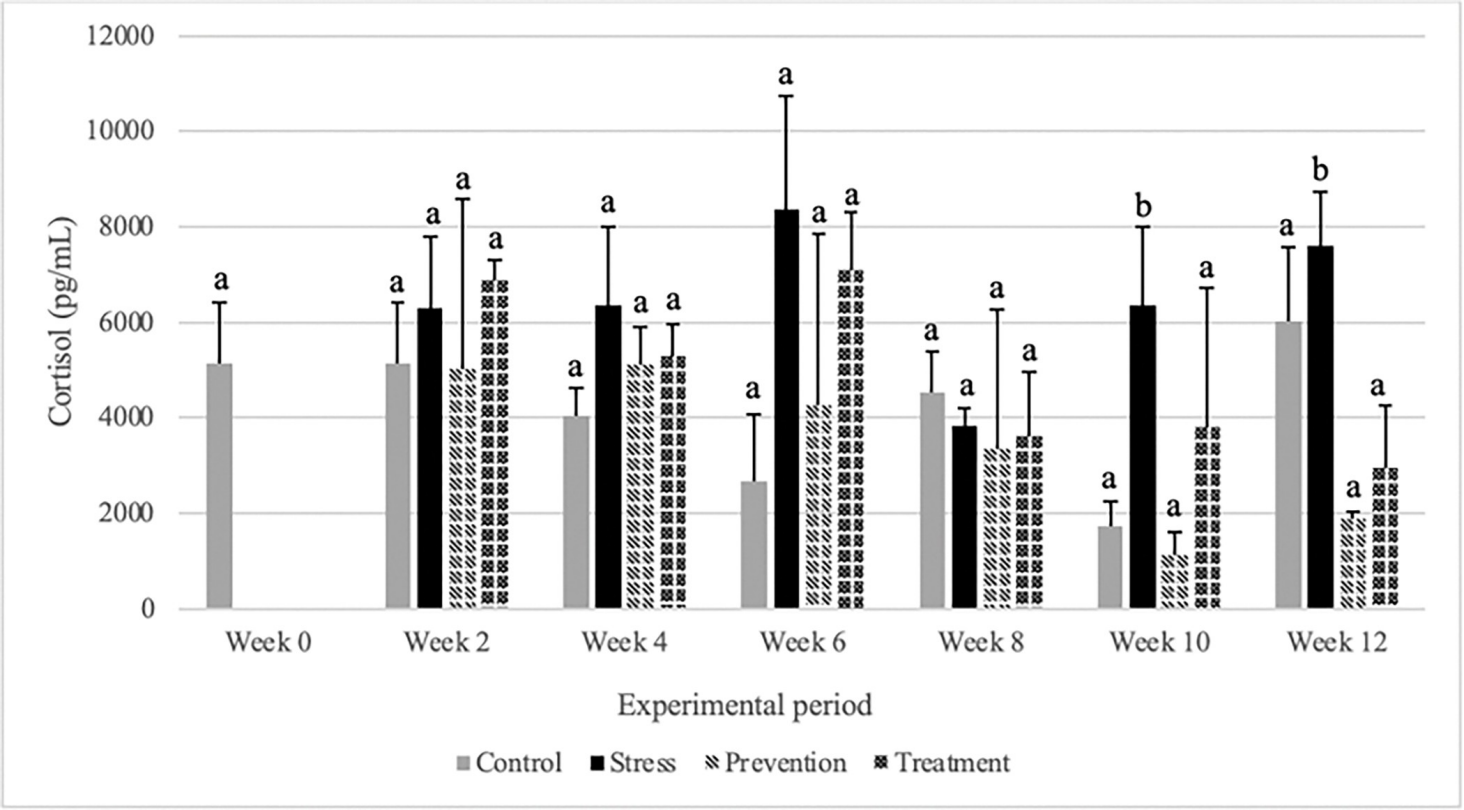
Plasma cortisol concentration in different experimental groups in tilapia. Concentrations (mean ± SD) are represented in groups: Control, stress, prevention and treatment. Different alphabets are significantly different than each other at a specific sampling period (*p*<0.05), n = 3.

After 12 weeks of experimentation, stress group showed a significantly higher cortisol level when compared to the control and prevention groups (n = 3; F = 15.778; p = 0.034) (Fig 2). At week 8 in (Fig 2), it has been seen that all the cortisol treated groups tried to acclimate. At week 10, the prevention group mitigated the release of cortisol significantly (n = 3; F = 5.699; p = 0.034) than the stress group. During our final week of experiment (week 12) the control group returned back to the baseline concentration.

**Fig 3:**
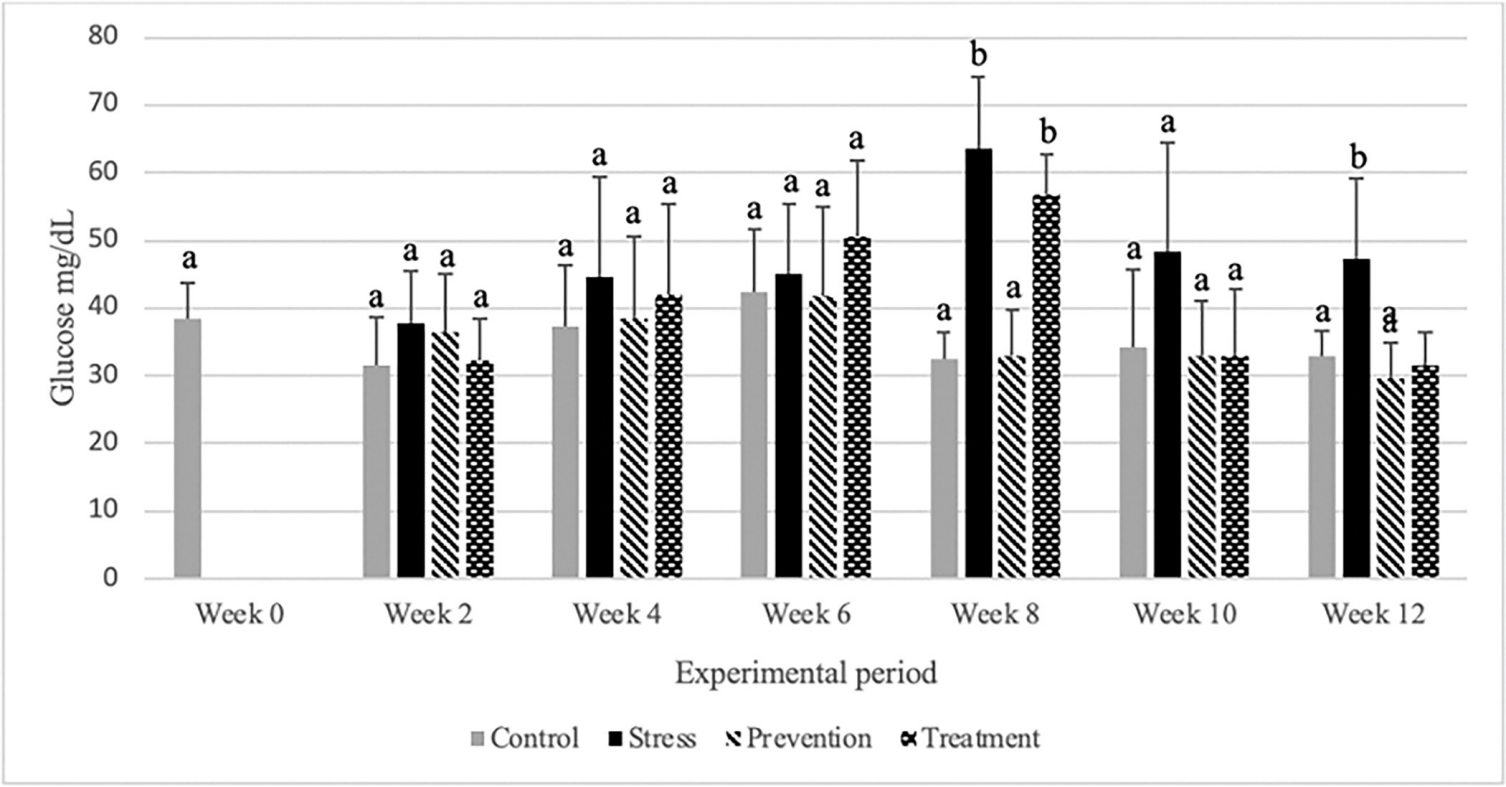
Blood glucose concentrations of tilapia in different experimental groups (control, stress, prevention, and treatment) were examined. The concentrations (mean ± SD) are presented in groups, and different letters indicate significant differences between groups at a specific sampling period (*p*<0.05), n = 6.

The prevention (n = 3; F = 15.778; p = 0.006) and the treatment (n = 3; F = 15.778; p = 0.002) groups were significantly lower than the stress group and the baseline concentration (Fig 3). The stress and treatment groups had over all higher glucose levels than the control and prevention groups (Fig 3). In week specific analysis, it was not significantly different from the comparable groups at 6 weeks (Fig 3). The prevention (n = 6; F = 7.703; p = 0.002) and treatment (n = 6; F = 7.703; p = 0.006) group successfully decreased the glucose level over the study duration of 12 weeks when compared to the stress group. Week 8 data revealed that the average glucose level was 63 mg/dL for the stress group while the levels of glucose for prevention and treatment groups were lowered to approximately 33 mg/dL. As the experiment progresses to week 12, glucose level representation maintained a similar pattern. In the last sampling day (12 weeks) the levels of glucose recorded for prevention and treatment groups were 29.5±5.3 mg/dL and 31.5±4.9 mg/dL, respectively, which were significantly lower than that of the stress group 47.3±11.7 mg/dL. In our experimental design the prevention group was treated with GPE from the very beginning of the experiment along with cortisol exposure. However, the treatment group started treatment with GPE after 6 weeks when its glucose level reached its peak. Despite the fact their starting time of the treatment is different, they showed similar results in terms of decreasing concentration of blood glucose. Therefore, after 12 weeks of repeated oral treatment, GPE has shown to be a nutraceutical that may be effective in lowering blood glucose levels.

**Fig 4:**
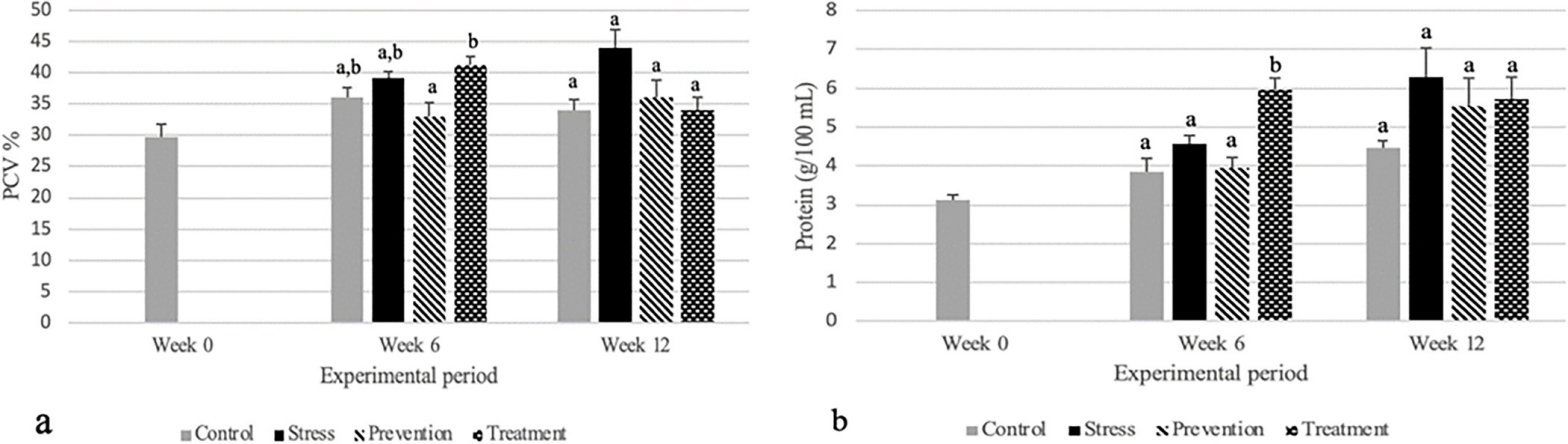
Packed cell volume (PCV) (a); plasma protein concentration (b) in tilapia in different experimental groups. Concentrations (mean ± SD) are represented in groups: Control, stress, prevention, and treatment. Different letters are significantly different than each other at a specific sampling period (*p*<0.05), n = 6.

In Fig 4A, at week 6 stress and treatment (also only treated with cortisol until 6 weeks) groups had the highest PCV. On the other hand, control and prevention groups had the lowest PCV with a significant difference between treatment and prevention groups at week 6 (n = 6; F = 5.389; p = 0.007). It can be stated that during primary responses of stress 2 of the 3 stress groups exhibited higher PCV. However, the prevention group was able to mitigate the stress as this group expressed lower PCV. We started to treat the treatment group at week 6 of sampling with the same amount of GPE as prevention group. Analyzing the week 12 data it can be said that the stress group remained higher, but the prevention and treatment groups showed lower PCV level than the stress group, even though not significantly different. In (Fig 4B), at week 6, we can see that all the control and prevention group exhibited the lower concentration of protein which is expected. Thus, in prevention group which was treated with GPE along with stress were able to mitigate the stress. Another 2 stress groups at 6 weeks should have released increased amount of protein, however the stress group did not as much as the treatment group released although they were treated exactly same until week 6. At week 6 treatment was significantly different from other 3 groups: control, stress and prevention (n = 6; F = 11.351; p<0.001).

**Fig 5:**
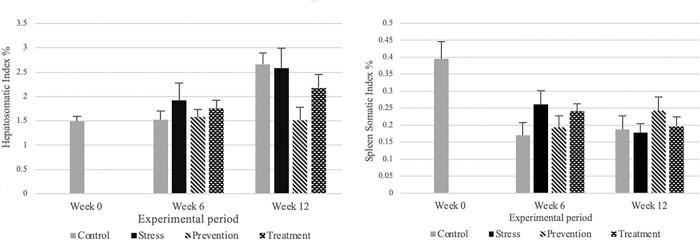
Hepatosomatic index % (a); Spleen somatic index % (b) in tilapia in different experimental groups. Concentrations (mean ± SD) are represented in groups: Control, stress, prevention, and treatment, n = 6.

Fig 5 [(Hepatosomatic index % (a); Spleen somatic index % (b)] exhibits that all the groups including control, stress, prevention, and treatment had no significant difference at weeks 6 and 12, for both HSI and SSII. The control group maintained a lower SSI at weeks 6 and 12. However, the stress group was higher at week 6 and lowest at week 12 which is expected when the stress is prolonged as at week 12 it reached an exhaustion phase. The prevention and treatment groups maintained the SSI at primary and secondary level of stress with the provided GPE treatment.

**Fig 6:**
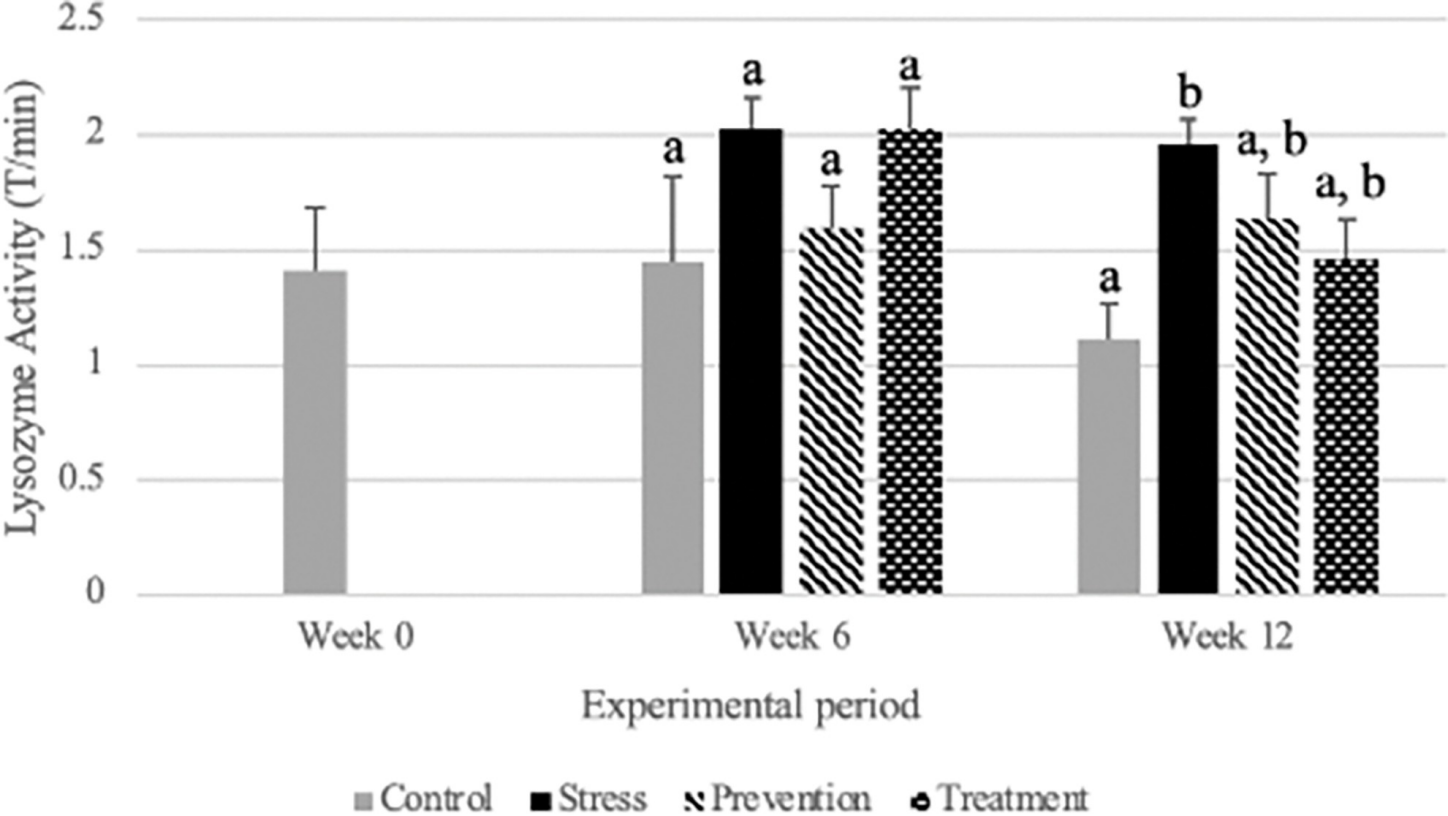
Lysozyme activity in different experimental groups in tilapia. Data (mean ± SD) are represented in groups: Control, stress, prevention, and treatment. Different letters are significantly different than each other at a specific sampling period (*p*<0.05), n = 6.

In Fig 6, where the stressed group had the highest transmittance value of 2.02 T/ min, higher than the control group at 1.45 T/ min. This difference is indicative of stress.

## Discussion

As of today’s knowledge, the effects of GPE on the stress physiology of fish is a new field of study. A handful of investigations have documented the effects of GPE on stress response in mice model. However, information was lacking for the fish model. Our research is fulfilling that gap by investigating the potential use of GPE as a stress-relieving agent in fish, specifically *O*. *niloticus* for the first time.

A cortisol assay was first carried out to see if the hydrocortisone supplied to the fish effectively and efficiently stressed the fish [31]. We required to understand how cortisol is produced in the body and how long it is active in order to fully comprehend the findings. Fish under stress produce adrenocorticotrophic hormones as a result of the hypothalamic-pituitary-interrenal axis being activated. Cortisol is consequently produced by the interrenal cells of the head kidney tissue. This is comparable to the adrenocorticotrophic hormone-stimulated cortisol production in animals, who also have a hypothalamic-pituitary-adrenal cortex [14]. In fish, levels of plasma cortisol recover to baseline 24 hours after an acute stressor is perceived and rapidly metabolized in the liver as a result of its action on it, before being filtered and eliminated from the body by the kidney [32]. In their experiment, Iskander *et al* demonstrated that hexane and toluene found from GPE extract inhibited hydrocortisone 4 mg/ear (mouse) at 35.0%. These tests revealed that steroids could be one type of anti-inflammatory compound found in this plant [33]. The cortisol concentration in plasma or serum is the most frequently measured indicator of the stress response in fish. The cortisol results from our study further support other studies that also found the cortisol concentration is increased in stress condition and remained unchanged till 24 hours [34–36].

In our study after 12 weeks, stress group showed higher level of cortisol then the prevention group. Prevention group had a cortisol level as low as control while the treatment group was in between control and the stress group. Specifically, up to 6 weeks we treated the stress treatment group with cortisol only. Therefore, the cortisol level of stress and treatment group was higher than the control group. Gynura species are known to possess anti-inflammatory effects. These anti-inflammatory properties work by modulation of inflammatory cytokine production, inhibition of prostaglandin E_2_ and nitric oxide production, cellular inflammatory-related parameters, and inflammation in animal models [37]. The results of the biological evaluation of GPE have supported the medical usage of this plant as a topical anti-inflammatory agent in Thai traditional medicine. Glucocorticoids are considered to be crucial stress hormones in vertebrates. Upon activation of the neuroendocrine stress axis, they are released into the bloodstream in response to real or perceived threats. Numerous studies have demonstrated that glucocorticoids have significant physiological effects on various target tissues [38]. As these effects assist vertebrates in counteracting the effects of stressors and preserving homeostasis, they are generally viewed as beneficial. Nonetheless, it is widely acknowledged that prolonged exposure to glucocorticoids can be harmful [39]. Glucocorticoids promote gluconeogenesis in the liver and decrease glucose uptake and utilization by antagonizing insulin response in skeletal muscle and white adipose tissue. Subsequently, excess glucocorticoid exposure causes hyperglycemia and insulin resistance. Glucocorticoids also regulate glycogen metabolism [40]. The average baseline glucose level found in our study was 38.51 mg/dL. This was congruent with the range reported by others, which is 34.54 and 130 mg/dL [41]. Algariri *et al* found that n-butanol fraction (n-BF) of ethanolic extract of Gynura showed the highest dose-dependent glucose-lowering action (51.2% and 62.0% at 500 mg/kg and 1000 mg/kg, respectively) in mice model, which is similar to metformin (63.6%, *p*<0.05). Moreover, they also found that GPE leaves have a no-observed-adverse-effect-level in mice [42]. The GPE group significantly reduced the increase in postprandial blood glucose levels compared to the control group of streptozotocin (STZ)-induced diabetic mice. In STZ-induced diabetic mice, GPE administration significantly reduced the area under the curve. These findings imply that GPE may aid in the treatment of postprandial hyperglycemia by inhibiting carbohydrate digesting enzymes [43].

Our observation agreed with the previous studies done on mice with GPE. In our experiment the effects on blood glucose were also studied following 12 weeks of repeated oral administration of the 0.15% GPE with 25% ethanol. Red blood cells perform major physiological and immunological functions in the body, making hematocrit or packed cell volume (PCV) a good indicator of physiological and immunological function. In stressful situations, there will be more blood cells to compensate for the increased demand on the body, resulting in a higher PCV [44]. Our findings also showed that the stress and treatment groups had the highest PCV at week 6 (they were also only treated with cortisol until six weeks). At week 6, however, there was a substantial difference between the treatment and prevention groups, with the control and preventive groups having the lowest PCV. At week 12, the stress group remained higher, but the prevention and treatment groups exhibited lower PCV levels than the stress group, even if there was no statistically significant difference between them. The plasma protein level is another important secondary stress biomarker. As previously stated, during stressed condition, plasma protein level get increased in animal body in order to supply proteins throughout the body to fix the damage of the tissues as a result of increased activity caused by stress [45]. It is expected that the level of protein should be higher in stressed animals. In our study we have observed significant higher amount of plasma protein concentration in treatment group at week 6 but week 12 data showed that there were no significant differences between groups. Nevertheless, prevention and treatment groups were released higher and lower amount of protein than the stress and control group respectively. The amount of protein is not always reliable to conclude the level of stress and physical condition. Iwama (1998) mentioned the effect of stress on plasma proteins. He pointed out some probable facts between stress and plasma protein. According to his findings plasma protein is not a suitable stress biomarker because of the wide variability in results [46].

Hepatosomatic index (HSI) is another secondary stress biomarker. HSI is the weight of the liver in proportion to the body weight. HSI should be low in animals undergoing acute stress due to the catabolism of glycogen found in liver. This breakdown allows for the increase in blood glucose, which is caused by acute stress [14]. This extra glucose is then used to meet the high energy demand that stress causes. However, with the exposure of chronic stress, glycogen stores in the liver as a form of energy. This increase in glycogen storage increases the liver weight and causes a new basal HSI. Chronic stress can lead to higher HSI as metabolic activity get increased in the liver. This trend is also found in earlier research [47]. Though we observed no statistical differences between groups in week 6 and 12 but prevention group showed a decreasing trend of HSI in both cases. This is suggestive of GPE’s capacity to lessen stress, but to the best of our knowledge, no study of a similar nature has been identified.

The weight of spleen in proportion to total body weight is considered as spleen somatic index which is a good biomarker since spleen is a reservoir for immune and red blood cells. Under stressed condition the spleen will release blood cells and splenic cells (T cells, B cells and macrophages) to compensate for increased respiratory demands and to fight off the perceived threat [29]. In the case of chronic stress, just like the liver, the spleen becomes larger to hold larger quantities of immune cells ready to be released in the case of another infection [48]. In research, published in 2017, found that mice exposed to chronic stress due to crowding were more likely to physically bite each other. The bite resulted in an increase in spleen weight within 19 days as the spleen’s immune cells became activated against the infection caused by the bites. [49]. However, an increase of immune cells may also affect the overall quality of the immune cells and their capacity to fight infections [50] though we observed no statistical significance among groups. Lysozyme activities are essential components of innate immunity. The hydrolysis of cell wall peptidoglycan is the canonical mechanism for bacterial killing by lysozyme. These defense mechanisms become activated during times of stress and express their secretory and cell membrane activity. A variety of stresses have the ability to affect them [51]. Studies reported, increased lysozyme activity and decreased other humoral immune components in tilapia associated with cadmium exposure. According to the study, pollutant exposure increases innate immunity while decreasing humoral immunity, allowing microbe susceptibility in polluted fish group [52]. Lysozyme activity is a tertiary stress biomarker that shows the ability of blood lysozyme to destroy bacteria in a solution. Clearer the solution, higher the amount of light that passes through it in comparison to the light emitted by the spectrophotometer. Therefore, clearer solutions will show a higher transmittance (T) reading. Higher lysozyme activity will be seen in stressed organism to fight against perceived infectious threat [53]. When there is more lysozyme in the blood, the amount of time taken to lyse present bacteria is decreased, therefore leading to higher transmittance values with an increase in lysozyme production. Our findings showed that stress group had the greatest transmittance value of 2.02 T/min, higher than the control group’s 1.45 T/min. Stress can be inferred from this variation. In a different study it was shown that handling stress in rainbow trout, *Oncorhynchus mykiss*, led to an increase in lysozyme activity [54]. Notably, at week 6, the prevention group showed reduced lysozyme activity when compared to the stress group which portray that GPE was able to reduce the lysozyme activity significantly from 2.02 to 1.5 T/ min; this is indicative of a reduction in stress. However, at week 12, with the introduction of the GPE treatment, the treatment group had lesser lysozyme activity than the stress group.

## Conclusion

Aquaculture’s role in providing a healthy, lean protein source for the world’s growing population is essential. Stress in fish is a significant issue due to the extensive husbandry methods commonly used in aquaculture. Overcrowding, mishandling, and vaccinating can cause fish stress. If exposure to stress is not limited or mitigated, stress will result in decreased productivity. Farmers frequently use antibiotics and chemical drugs to alleviate the symptoms that the stress response causes. When antibiotics and other chemical drugs are released into the environment, they can create superbugs or harm non-target species. Moreover, if the chemicals remain in fish body it may cause harm to human health. Using *Gynura procumbens* as functional feed could be a solution to the stress problem in the aquaculture industry. Functional feed, or nutraceuticals are not harmful to the animals, consumers, or the environment. Our experiment showed that cortisol level was significantly lower in prevention and treatment groups when compared to the stress group, which indicates that GPE was able to control chronic stress in Nile tilapia. Similarly, the glucose level of the stress group was also significantly higher than the control, prevention and treatment groups. All things considered, GPE, as a preventative treatment has the potential to reduce stress and improve immunity in fish. Since this is the first chronic stress study with GPE on fish, additional research with this plant could be beneficial in stress medication in aquatic organisms.

## Supporting information

S1 Data (XLSX)

S2 Data (XLSX)

## Abbreviations

GPE: *Gynua procumbens* Extract
HIS: Hepatosomatic index
SSI: Spleen somatic index
PCV: Packed cell volume
HPI: Hypothalamic-pituitary-interrenal axis
